# The effect of prey abundance and fisheries on the survival, reproduction, and social structure of killer whales (*Orcinus orca*) at subantarctic Marion Island

**DOI:** 10.1002/ece3.10144

**Published:** 2023-06-04

**Authors:** Rowan K. Jordaan, W. Chris Oosthuizen, Ryan R. Reisinger, P. J. Nico de Bruyn

**Affiliations:** ^1^ Mammal Research Institute, Department of Zoology and Entomology University of Pretoria Pretoria South Africa; ^2^ Centre for Statistics in Ecology, the Environment (SEEC) University of Cape Town Cape Town South Africa; ^3^ Ocean and Earth Science University of Southampton, National Oceanography Centre Southampton Southampton UK

**Keywords:** apex predator, environment, fishery, marine, population, threat

## Abstract

Most marine apex predators are keystone species that fundamentally influence their ecosystems through cascading top‐down processes. Reductions in worldwide predator abundances, attributed to environmental‐ and anthropogenic‐induced changes to prey availability and negative interactions with fisheries, can have far‐reaching ecosystem impacts. We tested whether the survival of killer whales (*Orcinus orca*) observed at Marion Island in the Southern Indian Ocean correlated with social structure and prey variables (direct measures of prey abundance, Patagonian toothfish fishery effort, and environmental proxies) using multistate models of capture–recapture data spanning 12 years (2006–2018). We also tested the effect of these same variables on killer whale social structure and reproduction measured over the same period. Indices of social structure had the strongest correlation with survival, with higher sociality associated with increased survival probability. Survival was also positively correlated with Patagonian toothfish fishing effort during the previous year, suggesting that fishery‐linked resource availability is an important determinant of survival. No correlation between survival and environmental proxies of prey abundance was found. At‐island prey availability influenced the social structure of Marion Island killer whales, but none of the variables explained variability in reproduction. Future increases in legal fishing activity may benefit this population of killer whales through the artificial provisioning of resources they provide.

## Graphical abstract

We tested whether the survival of killer whales (Orcinus orca) observed at Marion Island correlated with social structure and prey variables using multistate models of capture recapture data spanning 12 years. Survival was most strongly correlated with indices of social structure where higher sociality was associated with increased survival probability. Results also suggested that fishery‐linked resource availability is an important determinant of survival as survival.

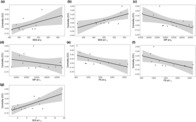

## 1 INTRODUCTION

Top predators strongly influence the structure and function of marine ecosystems (Estes et al., 2016; Estes & Duggins, 1995; Paine, 1980). However, due to environmental and anthropogenic changes, the global abundance of upper trophic‐level marine predators has declined (Estes et al., 2011; Hutchings & Baum, 2005). Many of these predators are keystone species, and declines in their abundance can trigger trophic cascades and downgrading of ecosystems (Estes et al., 2011; Pace et al., 1999). For example, diet switching by killer whales *Orcinus orca* in the Aleutian archipelago reduced sea otter *Enhydra lutris* population sizes, thereby releasing sea urchins from top‐down control and initiating a trophic cascade that transformed the kelp forest ecosystem (Estes et al., 1998). Given that marine predator declines (or in some cases population increases; Kitchell et al., 2006) can have far‐reaching ecosystem impacts through cascading top‐down processes, it is important to understand what environmental and anthropogenic factors regulate their population dynamics (Baum & Worm, 2009; Heithaus et al., 2008).

Bottom‐up (resource limitation) and top‐down (predation) mechanisms often act together to regulate the growth of animal populations (Leaper et al., 2006). Changes in resource availability due to environmental change are a major threat to the demographic resilience of many marine predators. If environmental change reduces prey populations, bottom‐up regulation of predator populations is likely to follow through changes in demographic parameters, including survival and reproductive rates (van den Hoff et al., 2014). Such environmentally driven population declines have occurred in many seal and seabird species inhabiting in the Southern Ocean (Weimerskirch et al., 2003). Human activities such as fisheries can exacerbate environmentally driven food limitation. African penguins *Spheniscus demersus*, for example, struggle to cope with recent shifts in the distribution of southern Benguela sardine *Sardinops sagax* and increased competition for food with purse‐seine fisheries, which leads to substantial adult mortality (Crawford, 1998; Sherley et al., 2014). Additionally, resource availability is linked to reproductive rates, with suppressed reproductive rates typical during periods of low prey availability (White & Ralls, 1993). In contrast, availability of anthropogenic food sources may increase reproductive output. For example, black bears *Ursus americanus* in urban areas with access to greater food availability have higher reproductive rates compared with bears in natural areas with lower food availability (Beckman & Lackey, 2008). Behavioral responses, including the use of anthropogenic resources and changes in social structure in social species, may thus allow predators to mitigate changes in prey abundances in some cases (Jordaan et al., 2021; Whitehead & Kahn, 1992).

The potential for conflict between marine predators and fisheries extends beyond prey depletion and competition for prey. Predators are, in fact, often attracted to fisheries by the foraging opportunities they provide, and some predators may benefit from interacting with fisheries (Barbraud et al., 2012). Typically, predators either take fish that have been caught in nets or by hooks (depredation) or they target escaped or discarded fish (Söffker et al., 2015; Tixier et al., 2020). For example, killer whales that depredate legal fisheries show increased survival and reproduction rates when compared to nondepredating individuals in the same population (Esteban et al., 2016; Tixier et al., 2015, 2017). But, many marine predators suffer from increased mortality due to direct interactions with fisheries (Carretta et al., 2019; Heithaus et al., 2008). These mortalities arise from animals being caught or entangled in fishing gear, or via retaliation from fishers that sometimes make use of firearms or explosives to repel predators (Jepsen & de Bruyn, 2019; Lewison et al., 2004).

Marine predator–fishery interactions that increase the mortality rates of predators may have major consequences for their population dynamics, especially when adults suffer increased mortality (Lebreton & Clobert, 1991). Additionally, fishery‐related mortalities may have knock‐on effects: Break up of pair‐bonds in wandering albatross *Diomedea exulans* reduces breeding success (Mills & Ryan, 2005) and disruption of the social structure of highly social top predators such as killer whales leads to prolonged demographic stress (Busson et al., 2019). Therefore, understanding fishery‐predator interactions, and the possible positive or negative effects on survival, reproduction, and social structure that arise from these interactions, is important.

Killer whales are long‐lived marine predators that occupy every ocean (Ford, 2009). As apex predators, they fulfill an important role in functioning ecosystems by regulating mesopredator populations (Estes et al., 1998). Killer whales, themselves, may be regulated by prey availability. Increases in natural prey (Chinook salmon *Oncorhynchus tshawytscha*), for example, are associated with increases in the survival of killer whales in the Eastern North Pacific (ENP; Ford et al., 2010). Furthermore, increases in fishery‐linked resource abundances (an “artificial” prey source) positively impact the survival of killer whales depredating Atlantic bluefin tuna *Thunnus thynnus* fisheries in the Strait of Gibraltar (Esteban et al., 2016) and legal longline Patagonian toothfish *Dissostichus eleginoides* fisheries in the Southern Indian Ocean (Tixier et al., 2017). In contrast, positive benefits of depredation are not associated with illegal fisheries; killer whales depredating illegal fishing vessels in the Southern Indian Ocean show decreased survival rates compared with nondepredating individuals (Guinet et al., 2015; Poncelet et al., 2010). These mortalities are known to have knock‐on effects that reduce the survival of remaining killer whales due to disruptions in their social structure (Busson et al., 2019).

Here, we investigate the behavioral and demographic responses of killer whales to environmental variation. Our analysis assessed (1) the relationship between survival and environmental, prey abundance, fisheries and social structure covariates, and (2) the relationship between social structure and reproduction, environmental, prey abundance, and fishery covariates. Our analysis is based on observation data obtained from an intensive long‐term, uninterrupted, photo‐identification study (2006–2018) at Marion Island in the Southern Indian Ocean. Specifically, we modeled the responses of killer whale survival to changes (immediate and lagged) in natural prey availability at Marion Island, and offshore estimates of both natural and “artificial” prey availability, including measures of Patagonian toothfish fishery effort. The response of social network measures and calving rate were modeled to the same prey and fishery variables. Social structure correlates with seasonal changes in prey abundances in this population (Jordaan et al., 2021), but whether killer whale survival correlates with social structure and/or longer‐term (interannual) variation in prey abundance is not known. In this context, we examine possible covariation between survival, prey abundance, and social structure. We predict that higher prey abundance and greater sociality (measured on the dyad and network level) will correlate with increased survival (Ford et al., 2010; Foster et al., 2012). As well as elucidating the response of killer whale survival to measures of prey availability, our results provide novel insight into how prey availability impacts the social structure and calving rate of this population of killer whales. In long‐lived species such as killer whales, social structure and calving rate are likely to exhibit more temporal variability than adult survival; these variables may therefore better reflect demographic responses to environmental variation compared with adult survival rates (Clements et al., 2022; Reid et al., 2005). Cumulatively, our results provide insight into the effects that changes in fisheries, environmental conditions, and social structure have on the behavior and demography of the apex predator in the Southern Indian Ocean.

## 2 METHODS

### 2.1 Study site

Marion Island (296 km^2^) and Prince Edward Island (45 km^2^) lie approximately 1800 km southeast of South Africa in the Southern Indian Ocean (46°54′ S, 37°45′ E). The two islands together form the Prince Edward Islands archipelago, an important breeding site for large populations of seals and seabirds (Ryan & Bester, 2008). Killer whales at Marion Island feed on a range of species including southern elephant seals *Mirounga leonina*, subantarctic fur seals *Arctocephalus tropicalis*, various penguins, Patagonian toothfish *Dissostichus eleginoides*, and possibly cephalopods (Reisinger et al., 2016; Reisinger, de Bruyn, Tosh, et al., 2011). The abundance of killer whales at the archipelago peaks during periods when inshore prey availability is high (predominantly during the elephant seal and penguin breeding seasons; Reisinger, de Bruyn, Tosh, et al., 2011). When not at the archipelago, these killer whales probably prey on Patagonian toothfish, potentially leading to interactions with commercial fishing vessels targeting the same species (Reisinger et al., 2015, 2016). Killer whale depredation of fish caught by long lines occurs in this area (Tixier et al., 2015, 2017; Williams et al., 2009), and some individuals that are part of the Marion Island population have been photographed from fishing vessels (Tixier et al., 2021).

### 2.2 Data collection and processing

Killer whale identification photographs were collected from shore at Marion Island from May 2006 to April 2018. Using various digital cameras and lenses, photographs were taken when killer whales were sighted by observers while doing other fieldwork (i.e., opportunistic sightings) or by observers conducting dedicated observation sessions. Dedicated observation sessions were conducted by trained observers who completed sessions of varying lengths (2–10 h) throughout the year at several locations of the island coastline most frequented by killer whales (Keith et al., 2001; Reisinger et al., 2015). During dedicated sessions, observers would remain at the same location and visually search for killer whales for the full, predetermined, session time. During all sightings, observers attempted to photograph the dorsal fin of each individual in the group and record the size of the group, its movement direction, and age/sex composition. Photographing continued until the group was out of photographic range.

Through careful examination of nicks, notches, and scratches on dorsal fins and saddle patches as well as the shape and form of dorsal fins, saddle patches, and eye patches (Bigg et al., 1987), individual killer whales were identified and matched to individuals in identification catalogs (Jordaan et al., 2019; Reisinger & de Bruyn, 2014). A quality score (ranging from 1 to 5 [unusable to excellent]) was assigned to all photographs. This score was based on the quality of lighting, focus and exposure and the size and level of obscurity of the dorsal fin in the photograph. Only sightings of individuals obtained from photographs with a quality score ≥ 3 were considered for analyses (Reisinger, de Bruyn, & Bester, 2011). Additionally, we excluded individuals (*n* = 15) that were seen less than four times during the study period in order to strengthen network analyses (Tosh et al., 2008).

### 2.3 Covariates influencing survival

#### 2.3.1 Direct measures of prey availability

**FIGURE A1:**
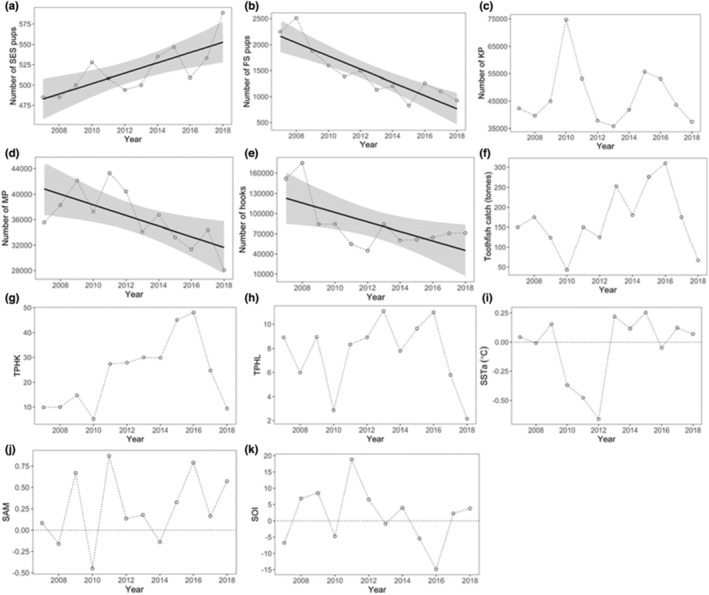
Temporal trends of covariates used to model the survival probability of killer whales at Marion Island from 2007 to 2018. Covariates included proxies of prey at Marion Island: (a) southern elephant seal pup numbers (SES), (b) subantarctic fur seal pup numbers (FS), (c) king penguin chick numbers (KP), and (d) macaroni penguin chick numbers (MP); measures of Patagonian toothfish fishing effort and catch: (e) total number of hooks set (TF), (f) the total catch (TFc), (g) the catch (tons) per 10,000 hooks (TPHK), and (h) the catch (tons) per haul (TPHL); and environmental conditions: (i) sea surface temperate anomaly (SSTa), (j) southern annular mode (SAM), and (k) Southern Oscillation Index (SOI). Regression lines (and 95% confidence intervals) are drawn for those covariates with statistically significant linear trends during the study period (Table A10).

Killer whale occurrence at Marion Island increases during seal (southern elephant seal and subantarctic fur seal) and penguin (king *Aptenodytes patagonicus* and macaroni penguin *Eudyptes chrysolophus*) breeding periods. Killer whales at Marion Island mostly prey on these four species when hunting inshore (Reisinger et al., 2016; Reisinger, de Bruyn, Tosh, et al., 2011), and thus, we predict that an increase in prey abundance would improve killer whale survival. This prediction assumes prey limitation and bottom‐up control of killer whale survival. We fitted annual counts of southern elephant seal pups (SES), subantarctic fur seal pups (FS), and king (KP) and macaroni (MP) penguin chicks as proxies of prey availability at Marion Island (Figure A1; Table A1). Island‐wide elephant seal pup counts were done on 15 October every year (Pistorius et al., 2011). Fur seal and penguin counts refer to counts of preweaning pups and prefledging chicks, respectively, done at selected study sites; these are assumed to represent the trends across the island (Wege et al., 2016, Department of Forestry, Fisheries and the Environment, unpublished data).

#### 2.3.2 Fishery covariates

The toothfish fishing industry around the Prince Edward Island archipelago (subarea 58.7 according to the Commission for the Conservation of Antarctic Marine Living Resources (CCAMLR)) is smaller than that at neighboring Îles Crozet (subarea 58.6), some ~900 km due east (4373 vs. 11,845 longline sets between 2006 and 2018; Tixier et al., 2020). Nevertheless, given that fishing vessels facilitate access to prey for killer whales through depredation, we predict that higher fishing effort by legal fisheries would correlate with increased killer whale survival. Yearly (May–April) fishery catch and effort data (CCAMLR, 2018) were collated, and four measures of fishing effort were fitted as covariates: the number of hooks set (TF), overall catch in tons (TFc), tons of catch per 10,000 hooks (TPHK), and the tons of catch per haul (TPHL). Fishery and direct measures of prey availability covariates were fitted with zero‐ to 3‐year time lags (t_0_, t_−1_, t_−2_ and t_−3_). Time lags were used to consider delayed impacts of prey availability on survival (Ford et al., 2005). Fishery data from subarea 58.6 (Îles Crozet) were not considered as only a small number of Marion Island killer whales have been seen in this area and movement between the two areas is not known to be frequent.

#### 2.3.3 Indirect proxies of prey availability

We used the Southern Oscillation Index (SOI), Southern Annular Mode (SAM), and sea surface temperature anomalies (SSTa) as indirect proxies of prey availability. SOI is an index of *El Niño*‐Southern Oscillation events, which result in changes in SSTa (Rasmusson & Wallace, 1983). SOI and SSTa provide indices of climatic and oceanographic variability over a small (SSTa) and large (SOI) scale and are closely associated with changes in marine food webs (Comiso et al., 1993; Croxall et al., 2002). SAM reflects extra‐tropical atmospheric variability in the Southern Hemisphere and, when positive, indicates a poleward shift in westerly winds that drive circulation of the Southern Ocean currents (Thompson & Wallace, 2000). Yearly (May to April) measures of these conditions were obtained. SST data were obtained for the geographical area frequented by killer whales when not at Marion Island, as determined from previous tracking data (Reisinger et al., 2015). This area (35–50° S; 30–44° E) is in South Africa's exclusive economic zone (EEZ) and within the CCAMLR Convention subarea 58.7 (CCAMLR, 2018). Averaged values of SOI (Commonwealth of Australia, 2020) were used with a 3‐ and 4‐year time lag as this is the time taken for oceanographic anomalies to form in this portion of the Southern Indian Ocean (Barbraud et al., 2008). SSTa (NOAA Physical Sciences Laboratory, 2020) values were calculated by subtracting the 5‐year running mean from the actual measured value for each month and then averaging across months. SAM was fitted with 0–2‐year time lags (t_0_, t_−1_ and t_−2_) to allow integration into the food web and intensification of the eddy field (Meredith & Hogg, 2006).

#### 2.3.4 Measures of social structure

Disruptions to killer whale social structure, for example, through mortalities arising from lethal responses by illegal fishers against depredating killer whales, reduce the survival probability of the remaining, closely associating, individuals (Busson et al., 2019). We therefore predict that higher social connectedness will correlate positively with the survival of Marion Island killer whales. Yearly (May to April) measures of social structure were calculated for each individual in the population and fitted in survival analysis as individual covariates. We considered the mean half‐weight index (HWI; an estimate of the proportion of time that two individuals spend together; Cairns & Schwager, 1987), the degree (DEGREE; the number of associations made), and mean centrality coefficient (CC; a measure of the broadness of the network; Beauchamp, 1965) as social structure covariates. These covariates were calculated in R 4.01 (R Core Team, 2020) with the use of the “asnipe” (Farine, 2019) and “igraph” (Csardi & Nepusz, 2006) packages (see Jordaan et al., 2021).

### 2.4 Covariates influencing social structure and reproduction

#### 2.4.1 Measures of prey abundance

Prey abundance and the presence and scale of fisheries may impact killer whale social structure (Foster et al., 2012; Jordaan et al., 2021) and reproduction (Tixier et al., 2015). We therefore assessed the temporal response of killer whale social network measures and reproduction to covariates of prey abundance and Patagonian toothfish fisheries. Prey abundance was represented by direct counts of prey availability at Marion Island (SES, FS, KP and MP) and indirect covariates of prey availability for at‐sea areas frequently visited by Marion Island killer whales (SOI, SAM, SSTa). Pup and chick counts are used as a proxy for the total population size of prey items (SES, FS, KP, and MP). Patagonian toothfish fishing catch and effort data (TF, TFc, TPHK, and TPHL) were used as covariates of fisheries.

These covariates were fitted with 0–1 (t_0_ and t_−1_) and 0‐ to 3‐year time lags (t_0_, t_−1_, t_−2_ and t_−3_) when testing their effect on measures of association and reproduction, respectively. The shorter (0–1 year) time lag was chosen as killer whale social structure at Marion Island is known to be fluid with observed differences between seasons (Jordaan et al., 2021). The longer (2–3 year) time lag was chosen for reproduction as this incorporates the 18‐month gestation period, 12‐month lactation period and the two‐year minimum calving interval for killer whales (Ford et al., 2005; Olesiuk et al., 2005; Tixier et al., 2015).

### 2.5 Data analysis

#### 2.5.1 Survival analysis

An encounter history matrix with 12 occasions was used to summarize individual identification data. Please see Jordaan et al., 2020 for details on data collection and processing as well as the age classes used. This study also showed similar sex‐specific survival between males and females for these data, and therefore, this was not considered for this study (Jordaan et al., 2020).

Data were analyzed in R 4.01 by calling MARK 8.0 (White & Burnham, 1999) through the RMark package (Laake, 2013). Program MARK makes use of multiple encounters of animals with artificial or natural markings and, through maximum likelihood methods, estimates survival and other population parameters (e.g., probability of transition between states). We constructed multiple competing models and ranked these using Akaike's Information Criterion corrected for small sample sizes (AICc). The model with the lowest AICc value represents the best compromise between model fit and complexity, with differences in AICc values (ΔAICc) indicating relative model support (Burnham & Anderson, 2002). Models received approximately equal support from the data if their ΔAICc scores were less than 2 units apart, though this is not a strict cutoff value (Burnham & Anderson, 2002). This approach of model selection assumes that the set of models included a general model that adequately fits the data. To verify this, goodness‐of‐fit testing was performed in U‐CARE 2.2.2 (Choquet et al., 2009) to test whether the Jolly‐MoVe (JMV) multistate model (Pradel et al., 2005) fitted the data. Homogeneous survival and detection probabilities among independently behaving marked animals are assumed in the JMV model (Pradel et al., 2005) in addition to the assumptions of capture–recapture models that marks are not lost, individuals are not misidentified, and sampling is instantaneous relative to the interval between occasions.

Using multistate capture–recapture models, we estimated survival (Φ), state transition (Ψ), and detection probabilities (*p*) of killer whales at Marion Island. We assumed time‐dependent detection and state‐dependent transition probabilities for all models, as these model structures were well supported in previous analysis of these data (Jordaan et al., 2020) and our interest was specifically on the survival parameter. For survival, we compared a model assuming constant survival (~1) to models with time‐ (~time), age class (~state), or covariate‐dependent survival. Covariates (Figure A1; Table A1) were added to test whether direct measures of prey availability (SES, FS, KP, and MP), measures of toothfish fishing effort (TF, TFc, TPHK, and TPHL), and indirect measures of prey availability (SOI, SAM, and SSTa) influenced survival between years. The significance of these covariates was evaluated using an analysis of deviance (ANODEV) test (Grosbois et al., 2008). Additionally, measures of social structure (DEGREE, HWI, and CC) were fitted as individual covariates to test whether survival probability varies as a function of social structure. All covariates (survival, social structure, and calving rate analyses) were standardized to mean = 0 and standard deviation = 1 to avoid numerical instabilities during analyses. Standardized covariates also allow for comparison of regression slopes between covariates that differ in order of magnitude (Schielzeth, 2010).

#### 2.5.2 Social structure and calving rate analysis

**FIGURE A2:**
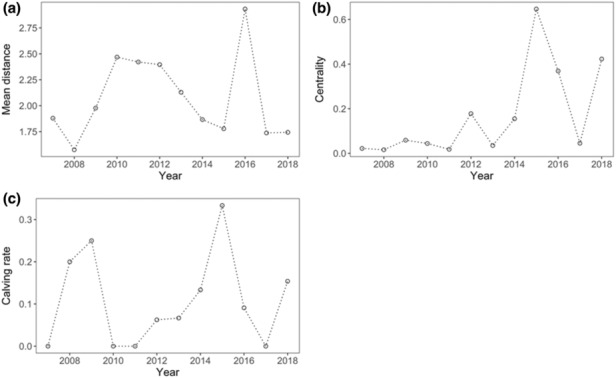
Temporal trends of population‐level social measures (a, b) and calving rate (c) of killer whales at Marion Island from 2006 to 2018. Social measures included: (a) mean distance (MD) and (b) centrality (CC).

Population‐level measures of association for weighted association networks were calculated in R 4.01 (R Core Team, 2020) with the “asnipe” (Farine, 2019) and “igraph” (Csardi & Nepusz, 2006) packages (see Jordaan et al., 2021). We calculated two measures of association between pairs of nodes (i.e., relationship measures): the mean distance between nodes (Mean distance) and centrality coefficient (CC; Figure A2a,b). Reproduction is represented by calving rate (the total number of calves born during a given year relative to the total number of “reproductively available” females in the population that year; Figure A2c; see Jordaan et al., 2020).

Linear mixed effects models with Gaussian error distributions (fitted using the “lme4” package (Bates et al., 2015) in R) were used to determine the relationship between response variables (Centrality, Mean distance, and calving rate) and covariates of interest. A set of models were constructed for each response variable, fitted with the “MuMIn” wrapper package (Bartoń, 2020) and ranked using the same AICc rules described previously. Covariates (Figure A1; Table A1) were added individually to test whether direct measures of prey availability (SES, FS, KP, and MP), measures of toothfish fishing effort (TF, TFc, TPHK, and TPHL), and indirect measures of prey availability (SOI, SAM, and SSTa) influenced response variables between years. An ANODEV test (Grosbois et al., 2008) was used to evaluate the significance of these covariates.

## 3 RESULTS

A total of 1997 dedicated killer whale observation sessions, totaling 11,194 h, were conducted at Marion Island from May 2006 to April 2018. During this time, 2668 sightings were recorded (0.24/h). An additional 2071 opportunistic sightings were recorded during the same period. A total of 89,792 identification photographs were taken of which 41,763 photographs from 2496 sightings were rated with a quality score ≥3.

From these, a total of 52 killer whales were identified (after exclusions), with 16 calves born into the population during this period. The encounter history data fitted the model assumptions according to goodness‐of‐fit test results, which showed nonsignificant results for component tests and the overall Jolly‐MoVe (JMV) model (Table A2).

### 3.1 Survival analysis

**FIGURE 1:**
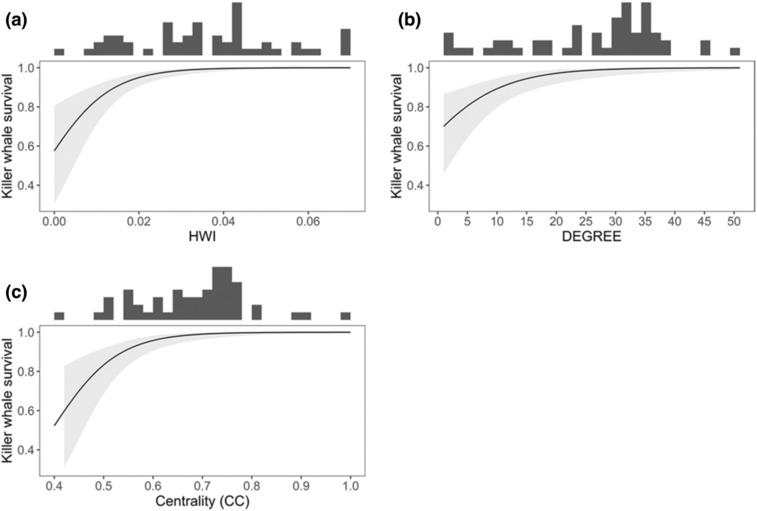
Marion Island killer whale survival as a function of social structure individual covariates (a) half‐weight index (HWI), (b) the degree, and (c) centrality in the same year. The shaded area represents the 95% confidence interval. The distributions of observed values of social structure are indicated by the histograms.

Multistate capture–recapture models with social structure indices as individual covariates were more parsimonious than those including prey, fishery, or environmental covariates. The model that included half‐weight index (HWI; the proportion of time that two individuals spend together) in the same year as an individual covariate was most parsimonious (Table 1). According to this model, survival averaged 0.991 (95% confidence interval (CI) = 0.972–0.997), with a significant positive relationship between survival and HWI (slope β = 2.20; 95% CI = 1.23–3.16; Figure 1a). Significant positive relationships were also present between survival and the social structure covariates DEGREE (β = 1.62 [95% CI = 0.72–2.52]) and CC (β = 1.56 [95% CI = 0.81–2.3]) in the same year (Figure 1b,c). However, these models were less well supported by the data (ΔAICc > 4).

**TABLE 1:** Model selection results for survival probability (Φ) obtained from multistate analysis of killer whale sighting histories at Marion Island (2006–2018).

Survival	K	ΔAICc	w_*i*_	−2lnL
~HWI	16	0.00	0.83	358.59
~DEGREE	16	4.24	0.10	362.83
~CC	16	5.10	0.07	363.69
~TF at t_−1_	16	24.43	0.00	383.01
~MP at t_0_	16	25.38	0.00	383.96
~FS at t_−1_	16	25.99	0.00	384.58
~TPHK at t_−1_	16	26.42	0.00	385.00
~FS at t_−3_	16	26.73	0.00	385.32
~SES at t_−3_	16	26.74	0.00	385.33
~FS at t_0_	16	26.92	0.00	385.51
~SAM at t_−1_	16	27.04	0.00	385.63
~1	15	27.04	0.00	387.80

*Note*: Omitted 32 models (total of 44 models). The number of parameters (K), ΔAICc (the difference in AICc between the model with the lowest AICc value and the relevant model) and AICc weight (*w*_*i*_) (the relative support of a model, in relation to the other models) and −2 log likelihood are presented. Models with ΔAICc values below that of the null model (~1) are presented (see Table A3 for all models fitted). All models assumed time‐dependent detection and state‐dependent transition from calf to juvenile to adult.

**FIGURE A3:**
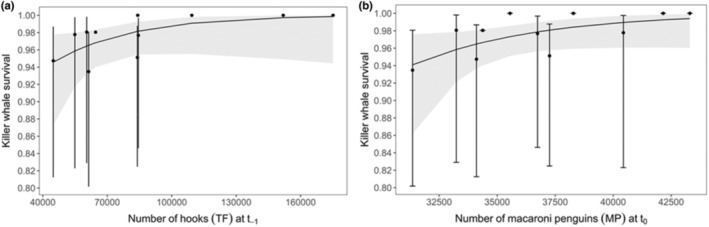
Marion Island killer whale survival as a function of (a) the total number of toothfish hooks set the previous year (TF at t_−1_) and (b) the total number of breeding macaroni penguins in the current year (MP at t_0_). The shaded area represents the 95% confidence interval. Point estimates for survival are from the most parsimonious fully time‐dependent model. Error bars depict 95% confidence intervals.

Models without individual covariates had no support in the data compared with those that included measures of social structure. Still, models where survival was constrained as a linear function of TF (the number of hooks set) at t_−1_ and MP (macaroni penguin chick counts) at t_0_ were 3.7 and 2.3 times better supported than the null model of constant survival (Table 1). These models showed that survival increased with the number of hooks set at t_−1_ (Figure A3a) and when there were more macaroni penguin chicks counted at t_0_ (Figure A3b) and explained 50.34% and 40.35% of the observed variability in survival, respectively (Table 2). The other covariates explained less of the variation in survival (their slope estimates were smaller and the 95% CI for β included zero; Table 2).

**TABLE 2:** Analysis of deviance (ANODEV) test results showing the effect of covariates on the survival probability of killer whales at Marion Island (2006–2018).

	Deviance_*F*,*df*_	*p*	Variation (%)	Slope (β) (95% CI)
Constant model	311.82_,15_			
Time‐dependent model	302.31_,25_			
Difference	9.51_,10_			
**Covariate: TF at t**_**−1**_	**307.03**_**9.12,16**_	**.01**	**50.34**	**1.13 (−0.19–2.45)**
**Covariate: MP at t**_**0**_	**307.98**_**6.09,16**_	**.04**	**40.35**	**0.87 (−0.07–1.82)**
Covariate: FS at t_−1_	308.6_4.61,16_	.06	33.86	0.71 (−0.12–1.55)
Covariate: TPHK at t_−1_	309.02_3.75,16_	.08	29.42	−0.52 (−1.13–0.08)
Covariate: FS at t_−3_	309.33_3.19,16_	.11	26.15	0.62 (−0.17–1.41)
Covariate: SES at t_−3_	309.34_3.17,16_	.11	26.05	−0.67 (−1.51–0.18)
Covariate: FS at t_0_	309.53_2.86,16_	.13	24.08	0.68 (−0.26–1.62)
Covariate: SAM at t_−1_	309.65_2.67,16_	.14	22.87	−0.51 (−1.18–0.16)
Covariate: SES at t_−2_	309.7_2.58,16_	.14	22.30	−0.50 (−1.16–0.16)
Covariate: SOI at t_−4_	309.85_2.35,16_	.16	20.73	−0.44 (−1.04–0.17)

*Note*: Omitted 28 covariates (total of 38 covariates). Deviance_*F*,*df*_ represents the deviance with the *F*‐statistic and the number of degrees of freedom. Variation (%) refers to the percentage variation of the deviance that is explained by a covariate. All significant covariates (*p* < .05) are presented in bold text. Only covariates with variation >20% are presented (see Table A1 for full test results).

**FIGURE A4:**
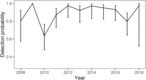
Annual probability of detection of killer whales at Marion Island from 2006 to 2018. Error bars depicting lower and upper confidence limits of the 95% confidence interval are included.

The probability of moving from the calf to juvenile state (Ψ = 0.36 [95% CI = 0.23–0.51]) was higher than the probability of moving from the juvenile to adult state (Ψ = 0.11 [0.06–0.20]). Detection probabilities varied annually (from 0.63 to 1) but were high overall (mean *p* = .91 [95% CI = 0.81–0.96]; Figure A4).

### 3.2 Reproduction and social structure analyses

Mean distance (the mean distance between nodes in the sociality matrix) showed weak relationships with SSTa and KP at t_−4_, but models incorporating these covariates were only marginally better (ΔAICc ranking) than the null model (Table 3), and ANODEV tests showed a nonsignificant effect on mean distance (Table A4).

**TABLE 3:** Model selection results for mean distance and centrality, population‐level measures of social structure, and covariates obtained from linear mixed effects models. Killer whale sighting histories at Marion Island (2006–2018) were analyzed to provide population‐level social measures.

*Response*	*Covariate*	*df*	ΔAICc	w_*i*_	logLik
Centrality	~SOI at t_−4_	3	0	0.451	−11.602
Centrality	~SES at t_−1_	3	2.43	0.134	−12.818
Centrality	~time	3	3.92	0.064	−13.561
Centrality	~SES at t_0_	3	3.98	0.062	−13.591
Mean distance	SSTa	3	0	0.18	−14.2
Mean distance	KP at t_−1_	3	0.81	0.12	−14.6
Mean distance	~1	2	1.03	0.11	−16.5
Mean distance	KP at t_0_	3	1.86	0.07	−15.1

*Note*: Omitted 19 models (total of 23 models) for both Mean Distance and Centrality. The number of degrees of freedom (*df*), ΔAICc (the difference in AICc between the model with the lowest AICc value and the relevant model) and AICc weight (*w*_*i*_) (the relative support of a model, in relation to the other models) and log likelihood (logLik) are presented. Models with ΔAICc values less than 2 (for Mean Distance) and 4 (Centrality) are presented (see Tables A8 and A9 for all models fitted).

**FIGURE 2:**
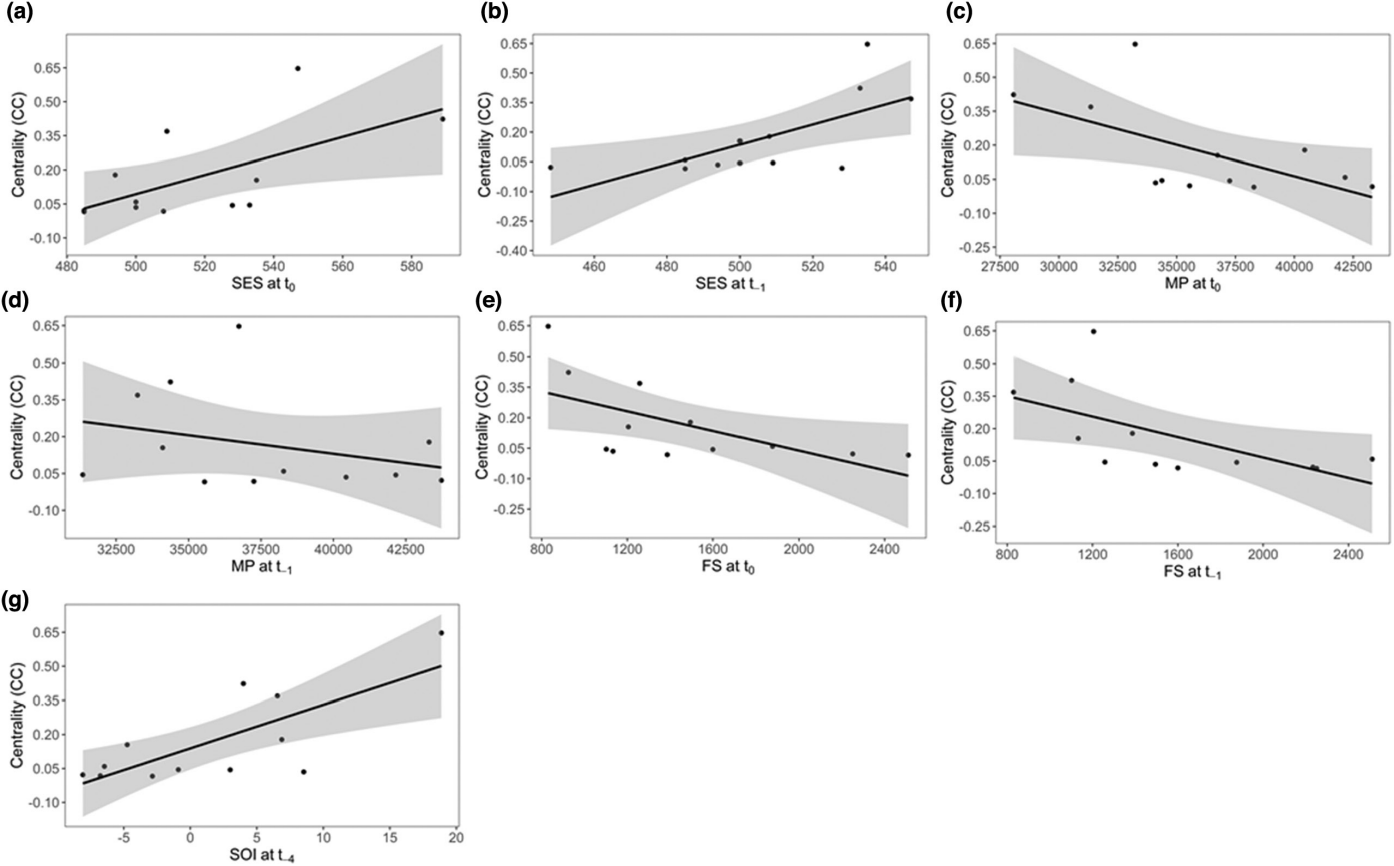
Marion Island killer whale population‐level centrality (CC) as a function of direct measures of prey availability at Marion Island (a–f) and indirect prey measures in the at‐sea foraging area of Marion Island killer whales (g). Centrality as a function of (a) southern elephant seal pup numbers in the current year (SES at t_0_), (b) southern elephant seal pup numbers 1 year previously (SES at t_−1_), (c) macaroni penguin numbers in the current year (MP at t_0_), (d) macaroni penguin numbers in the previous year (MP at t_−1_), (e) fur seal numbers in the current year (FS at t_0_), (f) fur seal numbers 1 year previously (FS at t_−1_), and (g) Southern Oscillation Index 4 years previously (SOI at t_−4_) are shown. All relationships are significant (see Table A6).

When investigating centrality (CC), the best‐supported model constrained centrality as a function of the Southern Oscillation Index 4 years previously (SOI at t_−4_) (Table 3). In addition, six other covariates, all direct measures of prey availability at Marion Island (SES, FS and MP), had a significant effect on centrality during the current (t_0_) and previous year (t_−1_) (Table 4). These effects were both positive (SES and SOI) and negative (FS and MP), suggesting that the Marion Island population of killer whales became less social as SES numbers increased but more social as FS and MP numbers increased (Figure 2).

**TABLE 4:** Analysis of deviance (ANODEV) test results showing the effect of covariates on the centrality of killer whales at Marion Island.

	Deviance_*F*,*df*_	*p*	Slope (β) (95% CI)
Constant model	9.93_,10_		
Time‐dependent model	7.12_,11_		
Difference	2.81_,1_		
**Covariate: SES at t**_**0**_	**6.776.25,10**	**.03**	**0.62 (0.13 to 0.88)**
**Covariate: SES at t**_**−1**_	**5.958.49,10**	**.02**	**0.68 (0.22 to 1.11)**
**Covariate: FS at t**_**0**_	**6.895.98,10**	**.03**	**−0.61 (−1.10 to −2.77)**
**Covariate: FS at t**_**−1**_	**6.856.05,10**	**.03**	**−0.61 (−1.10 to −2.78)**
**Covariate: MP at t**_**0**_	**7.045.62,10**	**.04**	**−0.60 (−1.10 to −2.75)**
**Covariate: MP at t**_**−1**_	**10.001.00,10**	**.34**	**−0.30 (−0.89 to −2.05)**
**Covariate: SOI at t**_**−4**_	**4.8612.64,10**	**.01**	**0.75 (0.34 to 1.40)**

*Note*: Deviance_*F*,*df*_ represents the deviance with the *F*‐statistic and the number of degrees of freedom. Only significant covariates (*p* < .05) are presented and are presented in bold text (see Table A6 for full test results).

**FIGURE A5:**
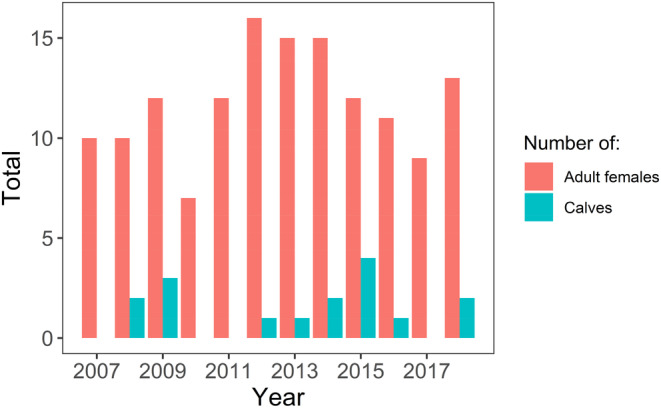
Number of reproductively available females and number of calves born to killer whales at Marion Island from 2006 to 2018. Females were deemed to be reproductively available if they were over the age of 10 years and did not have 1‐ or 2‐year‐old calves.

The number of calves born per year ranged from 0 to 4, while the number of reproductive females available for reproduction ranged from 7 to 16 individuals per year (Figure A5), equating to a mean calving rate of 0.11 (95% CI = 0.05–0.17) calves born per year per reproductive female (see also Jordaan et al., 2020). The model assuming constant calving rate over time was most parsimonious (Table 5). Although other models reflecting various measures of prey availability and fisheries covariates also received some support, none of the covariates had a statistically significant effect on calving rate (Table A5).

**TABLE 5:** Model selection results for the relationship between calving rate and covariates obtained from linear mixed effects models.

Response	Covariate	*df*	ΔAICc	w_*i*_	logLik
Calving rate	~1	2	0	0.08	−16.5
Calving rate	~TPHL at t_−2_	3	0.01	0.08	−14.7
Calving rate	~SSTa	3	0.13	0.08	−14.7
Calving rate	~TF at t_−3_	3	0.63	0.06	−15.0
Calving rate	~KP at t_−2_	3	0.99	0.05	−15.2
Calving rate	~KP at t_−1_	3	1.39	0.04	−15.4
Calving rate	~MP at t_−3_	3	1.47	0.04	−15.4
Calving rate	~SOI_−4_	3	1.71	0.03	−15.5

*Note*: Omitted 32 models (total of 40 models). The number of degrees of freedom (*df*), ΔAICc (the difference in AICc between the model with the lowest AICc value and the relevant model) and AICc weight (*w*_*i*_) (the relative support of a model, in relation to the other models) and log likelihood (logLik) are presented. Models with ΔAICc values <2 are presented (see Table A7 for all models fitted).

## 4 DISCUSSION

Survival of killer whales at Marion Island correlated with measures of social structure and fishery effort, but not with direct prey counts and indirect (environmental) proxies of prey availability. All measures of social structure showed a positive relationship with killer whale survival. Survival probability increased when killer whales spent more time with other individuals (HWI), associated with more individuals (DEGREE) and when the broadness of the network was reduced (CC). Survival was also positively correlated with toothfish fishing effort (but not catch, or catch per unit effort covariates) during the previous year. Furthermore, annual changes in at‐island prey availability were associated with changes in social structure of Marion Island killer whales. The social structure responded differently depending on prey type and the social structure index being considered. Reproduction was not influenced by any of the direct and indirect measures of prey. Together, these results show how important social structure is as a factor of survival in killer whales at Marion Island.

### 4.1 Fisheries and Marion Island killer whale survival

Prey availability (bottom‐up regulation) is an important determinant of animal survival (Hunt Jr & McKinnell, 2006). For example, Southern Resident killer whales in the ENP show survivorship trajectories that are strongly correlated with the availability of prey (Chinook salmon; Ford et al., 2010). We expected that fluctuating abundance of KP and SES at Marion Island may impact killer whale survival, considering that these prey species are presumed to be important dietary items here (Pistorius et al., 2012; Reisinger et al., 2016; Reisinger, de Bruyn, Tosh, et al., 2011). It is possible that the population sizes of these prey species are large enough for killer whales to exhibit a Holling type II functional response (Holling, 1959), leading to no impact on survival. This functional response occurs when predation has reached a saturation plane within which decreases or increases in prey density will not change predation rates. A Holling type II response will also mask the reproductive benefits of increased prey availability.

Satellite‐tracked killer whales that depart from Marion Island often move to seamounts north of the island, where they appear to forage on toothfish and possibly cephalopods (Reisinger et al., 2015, 2016). These seamounts are also frequented by fisheries targeting toothfish, placing Marion Island killer whales in close proximity of fishing vessels (Tixier et al., 2020). CCAMLR monitored toothfish fisheries in the area surrounding the archipelago lose 6% (15 tons) of their total annual catch to killer whale depredation (Tixier et al., 2020) providing an artificial food source. We detected no effect of fishery effort on social structure. In contrast, survival of Marion Island killer whales showed a positive relationship with the number of hooks deployed by fishing vessels during the previous year (TF at t_−1_; Figure A3a). An increase in fishing effort therefore correlates with higher Marion Island killer whale survival 1 year later. Apex predators are known to benefit from fisheries which aggregate or immobilize prey, increasing artificial resource availability. In the Strait of Gibraltar and off Îles Crozet, depredating killer whales show higher survival and fecundity rates compared with those that do not depredate (Esteban et al., 2016; Tixier et al., 2015, 2017). These benefits have likely resulted in an increase in the number of depredating killer whales in the waters surrounding Îles Crozet (Amelot et al., 2022; Tixier et al., 2019). The high survival of Marion Island killer whales suggests that any interactions that are occurring are probably with legal fisheries, where lethal responses by fishers to depredating killer whales are less likely. Alternatively, only a small proportion of the population are actively depredating. Future predicted expansions of fisheries may therefore have both positive and negative effects on killer whale survival depending on the scale and/or the presence of illegal fisheries. Depredation by Marion Island killer whales may not be widespread throughout the population as yet, but the known presence of some of its individuals at fishing vessels suggests that the number of depredating killer whales is likely to increase in the future (Amelot et al., 2022).

### 4.2 Sociality, reproduction, and prey abundance

Prey abundance is also an important driver of sociality in predators. The costs associated with living in groups are outweighed by its benefits when prey availability, and therefore individual energy gain, is greater (reviewed in MacDonald & Johnson, 2015). Sociality is therefore fluid and can vary over time in response to changes in prey availability. The Southern Resident killer whale population in the ENP, for example, is less social when their salmon prey availability is low and more social as prey availability increases (Foster et al., 2012). Here, we show that the link between prey availability and sociality is evident at Marion Island although the response of social structure is varied and dependent on prey type. This varied response in social structure is likely attributed to the generalist diet of this population (de Bruyn et al., 2013; Reisinger et al., 2015; Reisinger, de Bruyn, Tosh, et al., 2011). Previous work has shown that fission and fusion of killer whale social structure occur at Marion Island in response to seasonal changes in prey abundance (Jordaan et al., 2021). Sociality increases during periods of the year with greater prey abundance and decreases during winter, when prey is less abundant at the island (Jordaan et al., 2021).

We did not find relationships between prey availability and reproduction among Marion Island killer whales. Marion Island killer whales show reproduction rates closely resembling those of other global populations with differences among populations attributed to local ecology and stressors (Jordaan et al., 2020). Typically, resource availability is an important driver of reproduction in predator species with greater reproduction expected with increased prey availability through improvement of body condition (Brand & Keith, 1979). Killer whales at Îles Crozet and in the Strait of Gibraltar demonstrate this trend and show greater reproductive outputs when exposed to increased prey availability associated with fisheries (Esteban et al., 2016; Tixier et al., 2015). Social benefits may be the reason for the absence of a relationship between prey availability and reproduction, as observed here for Marion Island killer whales.

### 4.3 Limitations

Our analysis explored correlations between several response variables and a number of covariates, which increases the probability of a Type I error (i.e., that one or more of the covariates are significant due to chance; Gimenez & Barbraud, 2017). Gimenez and Barbraud (2017) suggest the use of a principal component analysis of covariates to resolve this issue. We performed a principal component analysis (results not shown) to reduce the number of covariates used in analysis, but none of the principal components were correlated with our response variables. Therefore, we decided to fit individual covariates in our models.

Another limitation is that we do not know how well our covariates reflect true prey availability to Marion Island killer whales and if the suit of covariates used covers all prey items of that this population feed on. These whales do not spend the entire year at the archipelago, and factors away from the island may thus also influence survival or social structure. For example, environmental proxies of prey abundance in the region of the seamounts did not correlate with killer whale survival, social structure, or reproduction. These environmental indices likely affect prey items at lower trophic levels, with the effects of these environmental factors taking time to reach apex predators like killer whales. However, the link between variability in climatic factors and foraging conditions and the impact these ultimately have on predators is not fully understood, particularly in the southern Indian Ocean (Pardo et al., 2017; Seyboth et al., 2016).

## 5 CONCLUSION

Artificial prey availability and social structure had the strongest correlation with the survival of Marion Island killer whales. Natural, inshore, prey availability was not suggested to impact survival with annual fluctuations in prey abundances potentially buffered by changes in social structure. This finding further strengthens support for social structure as an important modulator of survival in social apex predators. Future increases in legal fishing activity may prove to be beneficial to some apex predator populations, but the effects of these on the ecosystem and potential resource competition between fisheries and predators are not known (Mul et al., 2020). Uncertainty remains as to how variable climatic factors ultimately influence apex predators, but understanding these relationships is vital given current and predicted changes in climate conditions (Bestley et al., 2020; Convey & Peck, 2019).

## AUTHOR CONTRIBUTIONS

**Rowan Jordaan:** Conceptualization (supporting); data curation (lead); formal analysis (lead); writing – original draft (lead). **Chris Oosthuizen:** Formal analysis (supporting); methodology (supporting); supervision (equal); visualization (equal); writing – original draft (supporting); writing – review and editing (supporting). **Ryan Reisinger:** Conceptualization (equal); supervision (equal); visualization (equal); writing – original draft (supporting); writing – review and editing (supporting). **Nico de Bruyn:** Conceptualization (equal); funding acquisition (lead); project administration (lead); resources (lead); supervision (equal); writing – review and editing (supporting).

## CONFLICT OF INTEREST STATEMENT

The authors have no conflict of interest to declare.

## Data Availability

Data used for this manuscript can be found through the following DOI: https://doi.org/10.5061/dryad.905qfttr3.

## Appendix

**TABLE A1:** Analysis of deviance (ANODEV) test results showing the effect covariates on the survival probability of killer whales at Marion Island (2006–2018).

	Deviance_*F*,*df*_	*p*	Variation (%)	Coefficient (95% CI)
Constant model	311.82_,15_			
Time‐dependent model	302.31_,25_			
Difference	9.51_,10_			
Covariate: SES at t_0_	311.39_0.43,16_	.53	4.56	−0.33 (−1.32–0.65)
Covariate: SES at t_−1_	310.43_1.55,16_	.25	14.65	−0.47 (−1.25–0.31)
Covariate: SES at t_−2_	309.7_2.58,16_	.14	22.30	−0.50 (−1.16–0.16)
Covariate: SES at t_−3_	309.34_3.17,16_	.11	26.05	−0.67 (−1.51–0.18)
Covariate: FS at t_0_	309.53_2.86,16_	.13	24.08	0.68 (−0.26–1.62)
Covariate: FS at t_−1_	308.6_4.61,16_	.06	33.86	0.71 (−0.12–1.55)
Covariate: FS at t_−2_	310.48_1.48,16_	.25	14.14	0.42 (−0.27–1.12)
Covariate: FS at t_−3_	309.33_3.19,16_	.11	26.15	0.62 (−0.17–1.41)
Covariate: KP at t_0_	311.38_0.44,16_	.52	4.67	−0.21 (−0.80–0.38)
Covariate: KP at _t‐1_	311.79_0.03,16_	.87	0.29	0.06 (−0.68–0.81)
Covariate: KP at t_−2_	311.8_0.02,16_	.88	0.27	−0.05 (−0.68–0.58)
Covariate: KP at t_−3_	311.42_0.39,16_	.55	4.18	−0.20 (−0.82–0.42)
**Covariate: MP at t**_**0**_	**307.98**_**6.09,16**_	**.04**	**40.35**	**0.87 (−0.07–1.82)**
Covariate: MP at _t‐1_	311.82_0,16_	.95	0.05	0.03 (−0.84–0.90)
Covariate: MP at t_−2_	311.81_0.01,16_	.91	0.14	−0.05 (−0.90–0.80)
Covariate: MP at t_−3_	310.99_0.87,16_	.38	4.18	0.36 (−0.36–1.09)
Covariate: TF at t_0_	310.8_1.09,16_	.32	10.78	0.48 (−0.58–1.53)
**Covariate: TF at**_**t‐1**_	**307.03**_**9.12,16**_	**.01**	**50.34**	**1.13 (−0.19–2.45)**
Covariate: TF at t_−2_	311.09_0.75,16_	.41	7.66	0.36 (−0.46–1.18)
Covariate: TF at t_−3_	311.15_0.69,16_	.43	7.09	0.30 (−0.43–1.03)
Covariate: TFc at t_0_	310.76_1.13,16_	.31	11.19	−0.37 (−1.08–0.33)
Covariate: TFc at _t‐1_	311.32_0.5,16_	.50	5.23	−0.25 (−0.85–0.35)
Covariate: TFc at t_−2_	311.82_0,16_	.96	0.03	0.02 (−0.86–0.90)
Covariate: TFc at t_−3_	310.69_1.22,16_	.30	11.95	0.54 (−0.59–1.67)
Covariate: TPHK at t_0_	310.53_1.41,16_	.27	13.55	−0.42 (−1.13–0.30)
Covariate: TPHK at t_−1_	309.02_3.75,16_	.08	29.42	−0.52 (−1.13–0.08)
Covariate: TPHK at t_−2_	310.91_0.95,16_	.36	9.54	−0.39 (−1.11–0.34)
Covariate: TPHK at t_−3_	311.82_0,16_	.99	0.00	0.01 (−0.97–0.99)
Covariate: TPHL at t_0_	311.64_0.1,16_	.68	1.93	−0.21 (−1.23–0.80)
Covariate: TPHL at t_−1_	311.73_0,16_	.77	0.99	−0.12 (−0.87–0.63)
Covariate: TPHL at t_−2_	310.91_0.96,16_	.35	9.59	0.40 (−0.47–1.26)
Covariate: TPHL at t_−3_	310.88_0.99,16_	.35	9.89	0.38 (−0.45–1.21)
Covariate: SOI at t_−3_	311.29_0.53,16_	.49	5.55	0.27 (−0.47–1.00)
Covariate: SOI at t_−4_	309.85_2.35,16_	.16	20.73	−0.44 (−1.04–0.17)
Covariate: SSTa	311.82_0,16_	.96	0.03	−0.02 (−0.66–0.63)
Covariate: SAM at t_0_	311.62_0.19,16_	.67	2.10	0.18 (−0.65–1.02)
Covariate: SAM at t_−1_	309.65_2.67,16_	.14	22.87	−0.51 (−1.18–0.16)
Covariate: SAM at t_−2_	311.82_0,16_	.95	0.05	−0.03 (−0.80–0.75)

*Note*: Deviance_*F*,*df*_ represents the Deviance with the *F*‐statistic and the number of degrees of freedom. Variation (%) refers to the percentage variation of the deviance that is explained by a covariate. All significant covariates (*p* < .05) are presented in bold text.

**TABLE A2:** Goodness‐of‐fit test results for the Jolly‐MoVe (JMV) model fitted to multistate data of killer whales at Marion Island (2006–2018).

	χ^2^	*p*	*df*
Test 3G	11.79	.44	14
Test M	5.2	.13	4
JMV model	16.98	.41	18

*Note*: Test 3G tests for transience (i.e., differences in future encounter probabilities between newly identified and previously identified individuals). Test M tests for heterogeneity in detection (e.g., trap dependence—changes in detection following “captures” [i.e., sightings]).

**TABLE A3:** Full model selection results for survival probability (Φ), obtained from multistate analysis of killer whale sighting histories at Marion Island (2006–2018).

Survival	K	ΔAICc	*w*_*i*_	‐2lnL
~HWI	16	0.00	0.83	358.59
~DEGREE	16	4.24	0.10	362.83
~CC	16	5.10	0.07	363.69
~TF at t_−1_	16	24.43	0.00	383.01
~MP at t_0_	16	25.38	0.00	383.96
~FS at t_−1_	16	25.99	0.00	384.58
~TPHK at t_−1_	16	26.42	0.00	385.00
~FS at t_−3_	16	26.73	0.00	385.32
~SES at t_−3_	16	26.74	0.00	385.33
~FS at t_0_	16	26.92	0.00	385.51
~SAM at t_−1_	16	27.04	0.00	385.63
~1	15	27.04	0.00	387.80
~SES at t_−2_	16	27.09	0.00	385.68
~SOI at t_−4_	16	27.24	0.00	385.83
~SES at t_−1_	16	27.82	0.00	386.41
~FS at t_−2_	16	27.87	0.00	386.46
~TPHK at t_0_	16	27.93	0.00	386.51
~TFc at t_−3_	16	28.08	0.00	386.67
~TFc at t_0_	16	28.15	0.00	386.74
~TF at t_0_	16	28.19	0.00	386.78
~TPHL at t_−3_	16	28.27	0.00	386.86
~TPHL at t_−2_	16	28.30	0.00	386.89
~TPHK at t_−2_	16	28.31	0.00	386.90
~MP at t_−3_	16	28.38	0.00	386.97
~TF at t_−2_	16	28.49	0.00	387.07
~TF at t_−3_	16	28.54	0.00	387.13
~SOI at t_−3_	16	28.69	0.00	387.27
~TFc at t_−1_	16	28.72	0.00	387.31
~KP at t_0_	16	28.77	0.00	387.36
~SES at t_0_	16	28.78	0.00	387.37
~KP at t_−3_	16	28.82	0.00	387.41
~SAM at t_0_	16	29.01	0.00	387.60
~TPHL at t_0_	16	29.03	0.00	387.62
~TPHL at t_−1_	16	29.12	0.00	387.71
~KP at t_−1_	16	29.19	0.00	387.78
~KP at t_−2_	16	29.19	0.00	387.78
~MP at t_−2_	16	29.20	0.00	387.79
~SAM at t_−2_	16	29.21	0.00	387.80
~MP at t_−1_	16	29.21	0.00	387.80
~TFc at t_−2_	16	29.21	0.00	387.80
~SSTa	16	29.21	0.00	387.80
~TPHK at t_−3_	16	29.21	0.00	387.80
~state	17	31.10	0.00	387.50
~time	25	39.81	0.00	378.29

*Note*: The number of parameters (K), −2 log likelihood, ΔAICc (the difference in AICc between the model with the lowest AICc value and the relevant model), and AICc weight (*w*_*i*_) (the relative support by the data of a model, in relation to the other models) are presented. ~1 = constant, ~time = full time dependence and ~ state = age state. All models assumed time‐dependent detection and state‐dependent transition from calve to juvenile to adult.

**TABLE A4:** Full analysis of deviance (ANODEV) test results showing the effect of covariates on the calving rate of killer whales at Marion Island.

	Deviance_*F*,*df*_	*p*	Slope (β) (95% CI)
Constant model	11.00_,10_		
Time‐dependent model	10.9_,11_		
Difference	0.1_,1_		
Covariate: SES at t_0_	10.4_0.58,3_	.46	0.23 (−0.37–0.84)
Covariate: SES at t_−1_	10.560.42,10	.53	0.20 (−0.41–0.81)
Covariate: SES at t_−2_	9.661.39,10	.27	−0.35 (−0.93–0.23)
Covariate: SES at t_−3_	10.310.67,10	.43	−0.25 (−0.85–0.35)
Covariate: FS at t_0_	10.910.08,10	.78	−0.09 (−0.71–0.53)
Covariate: FS at t_−1_	10.970.02,10	.88	0.05 (−0.57–0.67)
Covariate: FS at t_−2_	10.830.15,10	.70	−0.12 (−0.74–0.49)
Covariate: FS at t_−3_	10.90.09,10	.77	−0.10 (−0.71–0.52)
Covariate: KP at t_0_	10.840.14,10	.71	−0.12 (−0.73–0.50)
Covariate: KP at t_−1_	9.12.09,10	.18	−0.42 (−0.98–0.15)
Covariate: KP at t_−2_	8.82.5,10	.14	−0.45 (−1.00–0.11)
Covariate: KP at t_−3_	10.80.19,10	.67	−0.14 (−0.75–0.48)
Covariate: MP at t_0_	10.850.14,10	.72	−0.12 (−0.73–0.50)
Covariate: MP at t_−1_	10.280.7,10	.42	−0.26 (−0.85–0.34)
Covariate: MP at t_−2_	10.720.27,10	.62	−0.16 (−0.77–0.45)
Covariate: MP at t_−3_	9.162.01,10	.19	0.41 (−0.16–0.97)
Covariate: TF at t_0_	10.990.01,10	.93	0.03 (−0.59–0.65)
Covariate: TF at t_−1_	9.791.24,10	.29	0.33 (−0.25–0.92)
Covariate: TF at t_−2_	110.001,10	.98	−0.01 (−0.63–0.61)
Covariate: TF at t_−3_	8.542.88,10	.12	−0.47 (−1.02–0.07)
Covariate: TFc at t_0_	10.130.86,10	.38	0.28 (−0.31–0.88)
Covariate: TFc at t_−1_	10.990.01,10	.93	0.03 (−0.59–0.65)
Covariate: TFc at t_−2_	10.360.62,10	.45	0.24 (−0.36–0.84)
Covariate: TFc at t_−3_	110.003,10	.96	0.02 (−0.60–0.64)
Covariate: TPHK at t_0_	10.420.57,10	.47	0.23 (−0.37–0.83)
Covariate: TPHK at t_−1_	10.890.10,10	.76	−0.10 (−0.72–0.52)
Covariate: TPHK at t_−2_	10.850.14,10	.71	0.12 (−0.50–0.73)
Covariate: TPHK at t_−3_	9.571.49,10	.25	0.36 (−0.22–0.94)
Covariate: TPHL at t_0_	10.790.2,10	.67	0.14 (−0.48–0.75)
Covariate: TPHL at t_−1_	9.991.01,10	.34	−0.30 (−0.89–0.29)
Covariate: TPHL at t_−2_	8.113.56,10	.09	0.51 (−0.02–1.04)
Covariate: TPHL at t_−3_	10.620.36,10	.56	0.19 (−0.42–0.80)
Covariate: SOI at t_−3_	10.810.18,10	.68	0.13 (−0.48–0.75)
Covariate: SOI at t_−4_	9.351.77,10	.21	0.39 (−0.18–0.96)
Covariate: SSTa	8.193.43,10	.09	0.51 (−0.03–1.04)
Covariate: SAM at t_0_	10.720.26,10	.62	0.16 (−0.45–0.77)
Covariate: SAM at t_−1_	9.91.11,10	.32	−0.32 (−0.90–0.27)
Covariate: SAM at t_−2_	10.790.19,10	.67	−0.14 (−0.75–0.48)

*Note*: Deviance_*F*,*df*_ represents the deviance with the *F*‐statistic and the number of degrees of freedom.

**TABLE A5:** Full analysis of deviance (ANODEV) test results showing the effect of covariates on the mean distance of killer whales at Marion Island.

	Deviance_*F*,*df*_	*p*	Slope (β) (95% CI)
Constant model	11.00_,10_		
Time‐dependent model	10.98_,11_		
Difference	0.02_,1_		
Covariate: SES at t_0_	10.370.61,10	.45	−0.24 (−0.84 to −1.89)
Covariate: SES at t_−1_	9.331.79,10	.21	0.39 (−0.18 to –0.03)
Covariate: FS at t_0_	10.770.22,10	.65	−0.15 (−0.76 to −1.63)
Covariate: FS at t_−1_	10.110.88,10	.37	−0.29 (−0.88 to −2.01)
Covariate: KP at t_0_	8.682.67,10	.13	0.46 (−0.09 to –0.28)
Covariate: KP at t_−1_	7.963.83,10	.08	0.53 (0.00 to –0.52)
Covariate: MP at t_0_	10.810.17,10	.69	0.13 (−0.48 to −0.82)
Covariate: MP at t_−1_	10.500.47,10	.51	0.21 (−0.39 to −0.56)
Covariate: TF at t_0_	8.802.51,10	.14	−0.45 (−1.00 to −2.41)
Covariate: TF at t_−1_	9.681.37,10	.27	−0.35 (−0.93 to −2.17)
Covariate: TFc at t_0_	10.700.28,10	.61	0.17 (−0.45 to −0.71)
Covariate: TFc at t_−1_	10.740.24,10	.63	−0.15 (−0.77 to −1.65)
Covariate: TPHK at t_0_	9.381.73,10	.22	0.38 (−0.19 to –0.01)
Covariate: TPHK at t_−1_	10.850.14,10	.71	0.12 (−0.50 to −0.86)
Covariate: TPHL at t_0_	9.861.16,10	.31	0.32 (−0.26 to −0.20)
Covariate: TPHL at t_−1_	10.640.34,10	.57	−0.18 (−0.79 to −1.73)
Covariate: SOI at t_−3_	11.000.00,10	.96	−0.02 (−0.64 to −1.26)
Covariate: SOI at t_−4_	10.770.22,10	.65	0.15 (−0.47 to −0.77)
Covariate: SSTa	7.444.80,10	.05	−0.57 (−1.08 to −2.68)
Covariate: SAM at t_0_	10.010.99,10	.34	0.30 (−0.29 to −0.27)
Covariate: SAM at t_−1_	10.520.45,10	.52	0.21 (−0.40 to −0.57)

*Note*: Deviance_*F*,*df*_ represents the deviance with the *F*‐statistic and the number of degrees of freedom.

**TABLE A6:** Full analysis of deviance (ANODEV) test results showing the effect of covariates on the centrality of killer whales at Marion Island.

	Deviance_*F*,*df*_	*p*	Slope (β) (95% CI)
Constant model	9.93_,10_		
Time‐dependent model	7.12_,11_		
Difference	2.81_,1_		
**Covariate: SES at t**_**0**_	**6.776.25,10**	**.03**	**0.62 (0.13 to 0.88)**
**Covariate: SES at t**_**−1**_	**5.958.49,10**	**.02**	**0.68 (0.22 to 1.11)**
**Covariate: FS at t**_**0**_	**6.895.98,10**	**.03**	**−0.61 (−1.10 to −2.77)**
**Covariate: FS at t**_**−1**_	**6.856.05,10**	**.03**	**−0.61 (−1.10 to −2.78)**
Covariate: KP at t_0_	10.890.10,10	.76	0.10 (−0.52 to **−**0.91)
Covariate: KP at t_−1_	10.930.06,10	.81	−0.08 (−0.70 to **−**1.44)
**Covariate: MP at t**_**0**_	**7.045.62,10**	**.04**	**−0.60 (−1.10 to −2.75)**
Covariate: TFc at t_0_	9.491.59,10	.24	0.37 (−0.21 to **−**0.03)
Covariate: TFc at t_−1_	10.520.46,10	.52	0.21 (−0.40 to **−**0.57)
Covariate: TPHK at t_0_	7.714.27,10	.07	0.55 (0.03 to 0.60)
Covariate: TPHK at t_−1_	9.291.84,10	.21	0.39 (−0.18 to 0.05)
Covariate: TPHL at t_0_	10.950.04,10	.84	0.07 (−0.55 to **−**1.02)
Covariate: TPHL at t_−1_	10.550.42,10	.53	−0.20 (−0.81 to **−**1.79)
Covariate: SOI at t_−3_	10.780.20,10	.66	0.14 (−0.47 to **−**0.79)
**Covariate: SOI at t**_**−4**_	**4.8612.64,10**	**.01**	**0.75 (0.34 to 1.40)**
Covariate: SSTa	10.260.72,10	.41	0.26 (−0.34 to **−**0.40)
Covariate: SAM at t_0_	9.931.08,10	.32	0.31 (−0.28 to **−**0.23)
Covariate: SAM at t_−1_	10.990.01,10	.91	−0.04 (−0.66 to **−**1.32)

*Note*: Deviance_*F*,*df*_ represents the deviance with the *F*‐statistic and the number of degrees of freedom. Significant covariates (*p* < 0.05) are presented in bold text.

**TABLE A7:** Full model selection results for the relationship between calving rate and covariates obtained from linear mixed effects models.

Response	Covariate	*df*	ΔAICc	w_*i*_	logLik
Calving rate	~1	2	0	0.08	−16.5
Calving rate	~TPHL at t_−2_	3	0.01	0.08	−14.7
Calving rate	~SSTa	3	0.13	0.08	−14.7
Calving rate	~TF at t_−3_	3	0.63	0.06	−15.0
Calving rate	~KP at t_−2_	3	0.99	0.05	−15.2
Calving rate	~KP at t_−1_	3	1.39	0.04	−15.4
Calving rate	~MP at t_−3_	3	1.47	0.04	−15.4
Calving rate	~SOI at t_−4_	3	1.71	0.03	−15.5
Calving rate	~TPHK at t_−3_	3	2	0.03	−15.7
Calving rate	~SES at t_−2_	3	2.1	0.03	−15.7
Calving rate	~TF at t_−1_	3	2.27	0.03	−15.8
Calving rate	~SAM at t_−1_	3	2.4	0.02	−15.9
Calving rate	~TPHL at t_−1_	3	2.51	0.02	−15.9
Calving rate	~TFc at t_0_	3	2.68	0.02	−16.0
Calving rate	~MP at t_−1_	3	2.86	0.02	−16.1
Calving rate	~SES at t_−3_	3	2.89	0.02	−16.1
Calving rate	~TFc at t_−2_	3	2.94	0.02	−16.1
Calving rate	~SES at t_0_	3	2.99	0.02	−16.2
Calving rate	~TPHK at t_0_	3	3.01	0.02	−16.2
Calving rate	~SES at t_−1_	3	3.17	0.02	−16.3
Calving rate	~TPHL at t_−3_	3	3.24	0.02	−16.3
Calving rate	~MP at t_−2_	3	3.35	0.02	−16.3
Calving rate	~SAM at t_0_	3	3.36	0.02	−16.4
Calving rate	~TPHL at t_0_	3	3.43	0.01	−16.4
Calving rate	~SAM at t_−2_	3	3.44	0.01	−16.4
Calving rate	~KP at t_−3_	3	3.44	0.01	−16.4
Calving rate	~SOI at t_−3_	3	3.45	0.01	−16.4
Calving rate	~FS at t_−2_	3	3.48	0.01	−16.4
Calving rate	~KP at t_0_	3	3.5	0.01	−16.4
Calving rate	~TPHK at t_−2_	3	3.5	0.01	−16.4
Calving rate	~MP at t_0_	3	3.5	0.01	−16.4
Calving rate	~TPHK at t_−1_	3	3.55	0.01	−16.4
Calving rate	~time	3	3.55	0.01	−16.4
Calving rate	~FS at t_−3_	3	3.56	0.01	−16.5
Calving rate	~FS at t_0_	3	3.57	0.01	−16.5
Calving rate	~FS at t_−1_	3	3.64	0.01	−16.5
Calving rate	~TF at t_0_	3	3.66	0.01	−16.5
Calving rate	~TFc at t_−1_	3	3.66	0.01	−16.5
Calving rate	~TFc at t_−3_	3	3.66	0.01	−16.5
Calving rate	~TF at t_−2_	3	3.67	0.01	−16.5

*Note*: Killer whale sighting histories at Marion Island (2006–2018) were analyzed to provide population‐level social measures. Calving rate was calculated as the number of calves born per year per reproductive female. The number of degrees of freedom (*df*), ΔAICc (the difference in AICc between the model with the lowest AICc value and the relevant model) and AICc weight (*w*_*i*_) (the relative support of a model, in relation to the other models) and log likelihood (logLik) are presented.

**TABLE A8:** Full model selection results for mean distance, a population‐level measure of social structure, and covariates obtained from linear mixed effects models.

Response	Covariate	*df*	ΔAICc	w_*i*_	logLik
Mean distance	SSTa	3	0	0.18	−14.2
Mean distance	KP at t_−1_	3	0.81	0.12	−14.6
Mean distance	~1	2	1.03	0.11	−16.5
Mean distance	KP at t_0_	3	1.86	0.07	−15.1
Mean distance	TF at t_0_	3	2.02	0.07	−15.2
Mean distance	SES at t_−1_	3	2.72	0.05	−15.5
Mean distance	TPHK at t_0_	3	2.79	0.04	−15.5
Mean distance	TF at t_−1_	3	3.16	0.04	−15.7
Mean distance	TPHL at t_0_	3	3.39	0.03	−15.8
Mean distance	SAM at t_0_	3	3.57	0.03	−15.9
Mean distance	FS at t_−1_	3	3.68	0.03	−16.0
Mean distance	SES at t_0_	3	3.99	0.02	−16.2
Mean distance	SAM at t_−1_	3	4.07	0.02	−16.19
Mean distance	MP at t_−1_	3	4.15	0.02	−16.2
Mean distance	TPHL at t_−1_	3	4.3	0.02	−16.3
Mean distance	TFc at t_0_	3	4.37	0.02	−16.3
Mean distance	TFc at t_−1_	3	4.42	0.02	−16.4
Mean distance	SOI at t_−4_	3	4.44	0.02	−16.4
Mean distance	FS at t_0_	3	4.44	0.02	−16.4
Mean distance	MP at t_0_	3	4.5	0.02	−16.4
Mean distance	TPHK at t_−1_	3	4.53	0.02	−16.4
Mean distance	~time	3	4.68	0.02	−16.5
Mean distance	SOI at t_−3_	3	4.7	0.02	−16.5

*Note*: Killer whale sighting histories at Marion Island (2006–2018) were analyzed to provide population‐level social measures. The number of degrees of freedom (*df*), ΔAICc (the difference in AICc between the model with the lowest AICc value and the relevant model) and AICc weight (*w*_*i*_) (the relative support of a model, in relation to the other models) and log likelihood (logLik) are presented.

**TABLE A9:** Full model selection results for centrality, a population‐level measure of social structure, and covariates obtained from linear mixed effects models.

Response	Covariate	*df*	ΔAICc	w_*i*_	logLik
Centrality	~SOI at t_−4_	3	0	0.451	−11.602
Centrality	~SES at t_−1_	3	2.43	0.134	−12.818
Centrality	~time	3	3.92	0.064	−13.561
Centrality	~SES at t_0_	3	3.98	0.062	−13.591
Centrality	~FS at t_−1_	3	4.13	0.057	−13.667
Centrality	~FS at t_0_	3	4.18	0.056	−13.693
Centrality	~MP at t_0_	3	4.45	0.049	−13.828
Centrality	~TPHK at t_0_	3	5.54	0.028	−14.371
Centrality	~1	2	6.14	0.021	−16.505
Centrality	~TF at t_0_	3	7.66	0.01	−15.432
Centrality	~TF at t_−1_	3	7.72	0.009	−15.462
Centrality	~TPHK at t_−1_	3	7.78	0.009	−15.494
Centrality	~TFc at t_0_	3	8.04	0.008	−15.622
Centrality	~SAM at t_0_	3	8.58	0.006	−15.891
Centrality	~MP at t_−1_	3	8.66	0.006	−15.933
Centrality	~SSTa	3	8.97	0.005	−16.085
Centrality	~TFc at t_−1_	3	9.27	0.004	−16.238
Centrality	~TPHL at t_−1_	3	9.31	0.004	−16.257
Centrality	~SOI at t_−3_	3	9.57	0.004	−16.386
Centrality	~KP at t_0_	3	9.68	0.004	−16.444
Centrality	~KP at t_−1_	3	9.73	0.003	−16.469
Centrality	~TPHL at t_0_	3	9.75	0.003	−16.479
Centrality	~SAM at t_−1_	3	9.79	0.003	−16.497

*Note*: Killer whale sighting histories at Marion Island (2006–2018) were analyzed to provide population‐level social measures. The number of degrees of freedom (*df*), ΔAICc (the difference in AICc between the model with the lowest AICc value and the relevant model) and AICc weight (*w*_*i*_) (the relative support of a model, in relation to the other models) and log likelihood (logLik) are presented.

**TABLE A10:** Linear temporal trends in at‐island prey, Patagonian toothfish fishing effort and environmental variables from 2006 to 2018. A t‐test was used to determine *p*‐values, testing the hypothesis that the slope of the relationship with the predictor equals zero.

Covariate	Coefficient ± SE	t‐value	*p*‐value	Adjusted *R* ^2^
**SES**	0.21 ± 0.06	3.68	**.00**	0.53
**FS**	−0.25 ± 0.04	−5.99	**.00**	0.76
KP	−0.03 ± 0.09	−0.34	.74	−0.09
**MP**	−0.19 ± 0.06	−2.94	**.01**	0.41
**TF**	−0.18 ± 0.07	−2.68	**.02**	0.36
TFc	0.08 ± 0.08	0.93	.38	−0.01
TPHK	0.14 ± 0.08	1.88	.09	0.19
TPHL	−0.03 ± 0.09	−0.38	.71	−0.08
SOI	0.06 ± 0.09	0.68	.52	−0.05
SSTa	0.07 ± 0.08	0.82	.43	−0.03
SAM	0.09 ± 0.08	1.03	.33	0.01

Abbreviations: FS, the number of subantarctic fur seal pups; KP, number of king penguins; MP, number of macaroni penguins; SAM, southern annular mode; SE, standard error; SES, the number of southern elephant seal pups; SOI, Southern Oscillation Index; SSTa, sea surface temperate anomaly; TF, total number of hooks set; TFc, total toothfish catch (tons); TPHK, tons of catch per 10,000 hooks set; TPHL, tons of catch per haul.

*Note*: When significant (*p* < .05), *p*‐values are presented in bold. *R* ^2^ is the coefficient of determination of the linear regression model.
